# Predicting involuntary admission among patients with psychotic disorder

**DOI:** 10.1192/j.eurpsy.2022.589

**Published:** 2022-09-01

**Authors:** E. Perfalk, L. Hansen, K. Nielbo, A. Danielsen, S. Dinesen

**Affiliations:** 1 Børglumvej 11, Department For Affective Disorders, Aarhus N, Denmark; 2 Aarhus University, School Of Culture And Society, Aarhus C, Denmark

**Keywords:** Precision Psychiatry, PSYCHOTIC DISORDERS, Involuntary admission

## Abstract

**Introduction:**

Involuntary admissions are increasing in numbers across Europe.^1^ They can be traumatic for the patients^2^ and are associated with large societal costs.^3^ Individuals with psychotic disorder are at particularly elevated risk of involuntary admission.

**Objectives:**

This study aims to investigate whether machine learning methods including natural language processing can predict involuntary admission among patients with psychotic disorder.

**Methods:**

We have obtained a dataset based on electronic health records for all patients having had at least one contact with the psychiatric services in the Central Denmark Region from 2011 to 2021. This dataset covers more than 120,000 patients, of which approximately 10,000 have been diagnosed with a psychotic disorder. The dataset contains both structured data, such as diagnoses, blood tests etc., as well as unstructured data (text). We will train machine learning models, basic logistic regression-models as well as state-of-the-art neural networks, to predict involuntary admission after contacts to the psychiatric services.

**Results:**

As the machine learning models are under development, no results are available at this time. Preliminary results are expected in spring 2022.

**Conclusions:**

If involuntary admission can be predicted among patients with psychotic disorder based on data from electronic health records, it will pave the way for potentially preventive interventions. References: 1. Sheridans-Rains, L et al., 2019 2. Frueh, B.C et al., 2005 3. Smith,S., 2020

**Disclosure:**

No significant relationships.

